# Teleost genomic repeat landscapes in light of diversification rates and ecology

**DOI:** 10.1186/s13100-023-00302-9

**Published:** 2023-10-03

**Authors:** William B. Reinar, Ole K. Tørresen, Alexander J. Nederbragt, Michael Matschiner, Sissel Jentoft, Kjetill S. Jakobsen

**Affiliations:** 1 Department of Biosciences, University of Oslo, Oslo, Norway; 2 Department of Informatics, University of Oslo, Oslo, Norway; 3 University of Oslo, Natural History Museum, Oslo, Norway

**Keywords:** Transposable elements, Short tandem repeats, Diversification, Repetitive DNA, Genome size, Genome dynamics

## Abstract

Repetitive DNA make up a considerable fraction of most eukaryotic genomes. In fish, transposable element (TE) activity has coincided with rapid species diversification. Here, we annotated the repetitive content in 100 genome assemblies, covering the major branches of the diverse lineage of teleost fish. We investigated if TE content correlates with family level net diversification rates and found support for a weak negative correlation. Further, we demonstrated that TE proportion correlates with genome size, but not to the proportion of short tandem repeats (STRs), which implies independent evolutionary paths. Marine and freshwater fish had large differences in STR content, with the most extreme propagation detected in the genomes of codfish species and Atlantic herring. Such a high density of STRs is likely to increase the mutational load, which we propose could be counterbalanced by high fecundity as seen in codfishes and herring.

**Supplementary Information:**

The online version contains supplementary material available at 10.1186/s13100-023-00302-9.

## Introduction

Repetitive sequences including transposable elements (TEs) and short tandem repeats (STRs) comprise large fractions of most eukaryotic genomes. STRs are repetitive stretches of DNA with unit sizes ranging from 1 to 10 bp, increasing and shrinking in size primarily due to replication slippage [1]. The origin of STRs in genomes has been attributed to processes of unequal crossing over [2], but STRs can also originate from parts of active TEs, as insertions of poly-A tails from retrotransposition, or from de novo mutations of STR-like patterns [3, 4]. TEs take advantage of the DNA replication and transcription processes of their hosts to facilitate propagation and are defined into two main classes: DNA transposons, which transpose directly from DNA to DNA, and retrotransposons (RTs) that transpose via an RNA intermediate. RTs are further divided into elements containing long terminal repeats (LTRs) and those that do not, the long interspersed nuclear elements (LINEs) and the short interspersed nuclear elements (SINEs) [5]. Both short and long tandem repeats have been shown to create genome challenges and errors at various levels in the sequencing-assembly-annotation-deposition workflow [6].

Comparative studies have revealed that transposable element (TE) content to some extent correlates with genome size variation across vertebrates [7] and across chordates [8]. Within more phylogenetically narrow taxa, differences in repeat content do not necessarily reflect the variation in genome size, such as within reptiles, mammals and birds [4, 9]. In the largest vertebrate group, teleost fish, the correlation between genome size and repetitive DNA content appears to be modest [7, 8, 10–12], with the largest study [12] reporting an R of 0.6 (R^2^: 0.36). In contrast, TE content has been suggested to explain 98% of the variation in genome size in angiosperms [13]. Due to the nature of TE propagation, it is not surprising that an increase in TE copies may lead to an increase in genome size. The empirical evidence is, however, less clear for correlations between STR content and genome size. Across eukaryotic kingdoms, the relationship between STR content and genome size seems to be positive [14–16], whereas, no significant correlation has been reported within kingdoms [17]. Intriguingly, for teleost fish, genome size seems to be linked to differences in egg diameter, parental care and aquatic habitat (saltwater or freshwater) Hardie and Hebert [18]. These factors have so far not been taken into account when testing the relationship between genome size and repetitive DNA in teleosts.

Beyond their contribution to genome size variability, TEs have been postulated to cause deletions, translocations, duplications, and inversions in response to stress conditions [19, 20]. For instance, TEs has been indicated to be of evolutionary importance in invasive species of ants, where TE-dense genomic islands were shown to generate variability in genes deemed important in the adaptation process [21]. Interestingly, bursts of TE activity coinciding with speciation have been found in studies of a variety of taxa [22], including mammals [23]. Within teleost fish, elevated TE activity has been shown to coincide with species radiations in salmonids [24] and cichlids [25, 26]. Beside a potential role in adaptive radiations through generating adaptive mutations, a mechanism of which TEs could influence speciation is by causing chromosomal rearrangements, possibly as a response to epigenetic release due to environmental stress [22], which in turn can lead to reproductive isolation. For STRs, different length variants present in a population contribute to the genetic variation and have been shown in some cases to be functionally relevant [27–30]. As with TEs, STR content varies across vertebrates, with frequencies from approximately 100 loci/Mbp to 1000 loci/Mbp and densities from 1000 bp/Mbp to 50 000 bp/Mbp [31–33]. A large proportion of these STRs occur outside genes; however, in humans for instance, around 4500 STRs occur in protein coding regions [34]. A STR within an open reading frame (ORF) often encodes homo- or di-amino acid tracts that to a large extent overlap with intrinsically unstructured protein regions [35, 36]. Such regions are abundant in proteins that interact with other proteins [37]. On the other hand, STRs occurring in regulatory regions can affect the expression of genes [30, 38, 39] and STRs in introns may impact RNA splicing [29, 40].

In light of the above-mentioned observations, a key question is to what extent the genomic repeat landscape impacts the evolution of teleost fishes. First, we investigated the interplay between genome size, aquatic habitat, parental care, and repetitive DNA content, using comparative methods taking phylogenetic relationships as well as assembly quality into account. Next, we focused on diversification. Our focal group, teleosts, is the most species rich group of all vertebrates and serves as a suitable system to test for associations between the TE/STR landscape and diversification, given the genomic sequencing initiatives of multiple teleost species [41–43] as well as available species richness data. Teleostean families differ widely in species diversity, ranging from monotypic families such as Helostomatidae to the Cyprinidae, with ~ 3,000 species. Estimates of the percentage of TEs in teleost genomes vary from 6–7% (Tetraodon) to 55–56% (zebrafish) [7, 11], and estimates of the number of STR loci range from 1,180 loci per Mbp in Atlantic cod to 219 loci per Mbp in medaka [33]. We annotated the TE and STR content in the genome assemblies of 100 teleost fish (41 taxonomic orders and 70 families) and one non-teleost ray-finned fish (spotted gar, *Lepisosteus oculatus*). Our samples cover the major teleost branches, allowing us to describe differences in TE and STR content after ~ 270 million years of evolution, and to investigate the role of repetitive DNA in teleost genome size evolution and its potential influence on diversification.

## Results

### TE count variation consistent between read-based and assembly-based methods

**Fig. 1:**
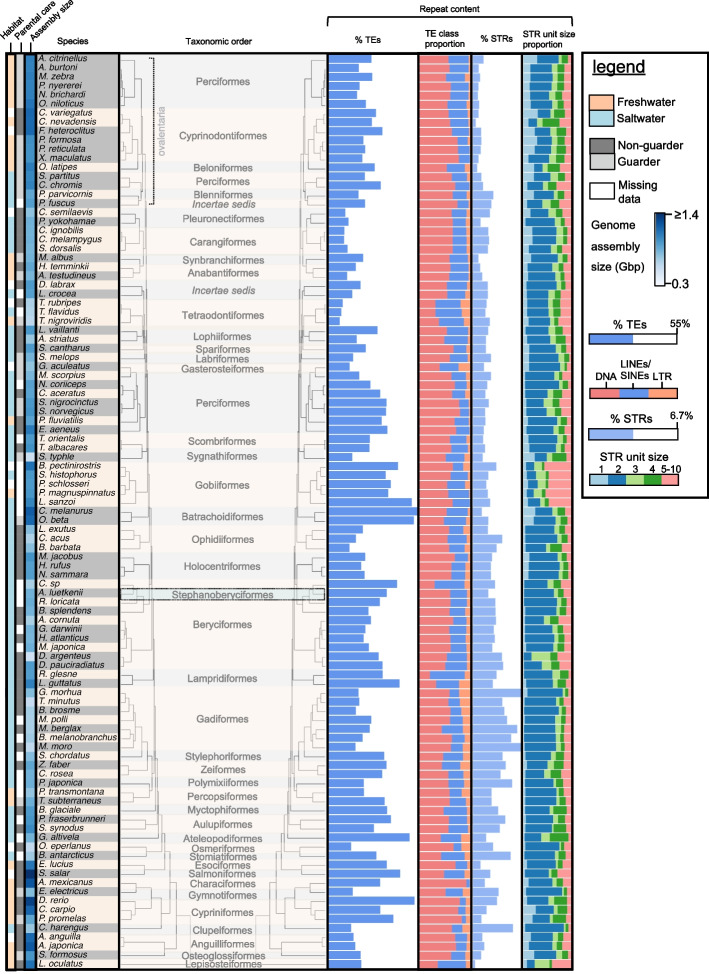
Genome assembly sizes, ecological variables (habitat, parental care) and the repetitive DNA content in 101 fish genomes. The phylogenetic tree was retrieved from [43]. Species names and taxonomic orders are indicated. Species belonging to the same taxonomic family share colors of beige and gray. The values of ecological variables ("habitat" and "parental care") are indicated with color: Freshwater (orange), saltwater (blue), non-guarding behavior (dark gray), guarding or bearing behavior (light gray) (see legend). Genome assembly size ("assembly size") is indicated by shades of blue (see legend). For clarity, the genome assembly size maximum was set to 1.4 Gbp. The genomic percentage of TEs ("% TE"s) are shown by blue bars. The longest bar (*C. melanurus*) represents 55% TEs and the shortest (*T. nigroviridis*) represents 6.7% TEs (see legend). Stacked colored bars ("TE class proportion") show the relative proportions of TEs; DNA transposons (red), LINEs and SINEs (blue) and LTR retrotransposons (orange). The genomic percentage of STRs ("% STRs") are shown by blue bars. The largest bar (*G. morhua*) represents 6.7% STRs and the shortest (*L. oculatus*) represents 0.3% TEs (see legend). Supplementary Table 1 contains the source data

We ran an assembly-specific TE discovery pipeline on each assembly (see Materials and Methods). We treated the percentage of total interspersed repeats (i.e., the number of bases in an interspersed repeat divided by the number of bases in the assembly), which included both classified and unclassified interspersed elements as a proxy for TE-content, because the total interspersed repeat counts are not biased by homology-based TE classification and were strongly correlated with the number of interspersed elements classified as TEs (R^2^: 0.67, Supplementary Fig. 1a). However, we note that the estimated fraction of each TE class likely suffers from the 'classification-by-homology' bias ("TE class proportion", Fig. 1). The quality of the genome assemblies ranged from fragmented (lowest contig N50: 1,119 bp for *Takifugu flavidus*) to contiguous (highest contig N50: 10,734,51 bp for *Danio rerio*) (Supplementary Table 1) and could bias the discovery of longer TEs. We compared estimates of TE content between an assembly-based approach and a read-based approach for 53 of the 101 genome assemblies, and found that, in these assemblies, the percentage of TEs detected in the assemblies correlated with the percentage detected in the reads used to generate the assembly (R^2^: 0.82, mean difference: 0.76%, SD: 7.2%) (Supplementary Fig. 1b). Given the strong correlation, we used the assembly-based annotation results in further analyses.

### Substantial changes in TE content over 270 million years of evolution

Summary of the annotation of repetitive DNA in teleost genomes and the ecological variables we gathered for each species, as well as the genome assembly sizes is presented in Fig. 1. There was substantial variation among species in terms of TE content, STR content, and genome assembly sizes across the teleosts (Fig. 1). The DNA transposon content ranges from 1.6% in tetraodon (*Tetraodon nigroviridis*) to 37.1% in zebrafish (*Danio rerio*). The LTR-RT content ranges from 0.48% in bluefin trevally, southern platyfish and climbing perch (*Caranx melampygus*, *Xiphoporus maculatus* and *Anabas testudineus*) to 7.4% in opah (*Lampris guttatus*). LINE content varies from 0.89% in blind cavefish (*Astyanax mexicanus*) to 12.6% in giant oarfish (*Regalecus glesne*) and SINE content ranges from 0.02% in electric eel (*Electrophorus electricus*) to 3.6% in giant oarfish (*R. glesne*) (Fig. 1). We quantified the proportions of DNA transposons, LTR retrotransposons, LINEs and SINEs relative to the total classified TE content ("TE class proportion"). We find that DNA transposons collectively make up the largest proportion of the TE composition in most teleost fish genomes (94 out of 101 species, Fig. 1). However, we find multiple lineage-specific differences in TE composition. The DNA transposon fraction seem especially high in Characidae (*Astyanax mexicanus*: 89.7%), Cyprinidae (mean: 77.5%, SD: 2.8%), Sebastidae (mean: 76.9%, SD: 0.8%) and Poeciliidae (mean: 74.1%, SD: 1.1%). Of retrotransposons, LINEs are the most prevalent TE subclass, and display the highest relative fractions in *Cetomimus* sp. (51.4%), *Regalecus glesne* (51.0%), Tetraodontidae (mean: 44.9%, SD: 0.9%) and *Lampris guttatus* (40.9%). The LTR-RT fraction is comparably low in most of the genomes studied, but is largest in *Gasterosteus aculeatus* (25.2%), *L. guttatus* (24.2%) and Gadidae (mean: 23.3%, SD: 0.7%). Relative SINE fractions are generally low (mean: 4.1%, SD: 0.3%), with exceptions being *Synodus synodus* (16.8%), the non-teleost *Lepisosteus oculatus* (16.5%) and *R. glesne* (14.5%). The Tetraodontidae family (represented by *Takifugu rubripes*, *Takifugu flavidus* and *Tetraodon nigroviridis*) have a particularly small fraction of DNA transposons (mean: 33.5%, SD: 3.1%), a feature shared only with distant relatives such as *Cetomimus sp.*, *L. oculatus* and *R. glesne*. The two lampriform fishes (*R. glesne* and *L. guttatus*) stand out from other fishes in TE composition in that the lampriformes have a low relative fraction of DNA transposons (and higher fraction of LINEs/SINEs). Overall, the large differences in TE composition among and sometimes within teleost families highlight the dynamic nature of TEs during teleost evolution.

### Interplay between genome size, repetitive DNA and ecological factors

**Fig. 2:**
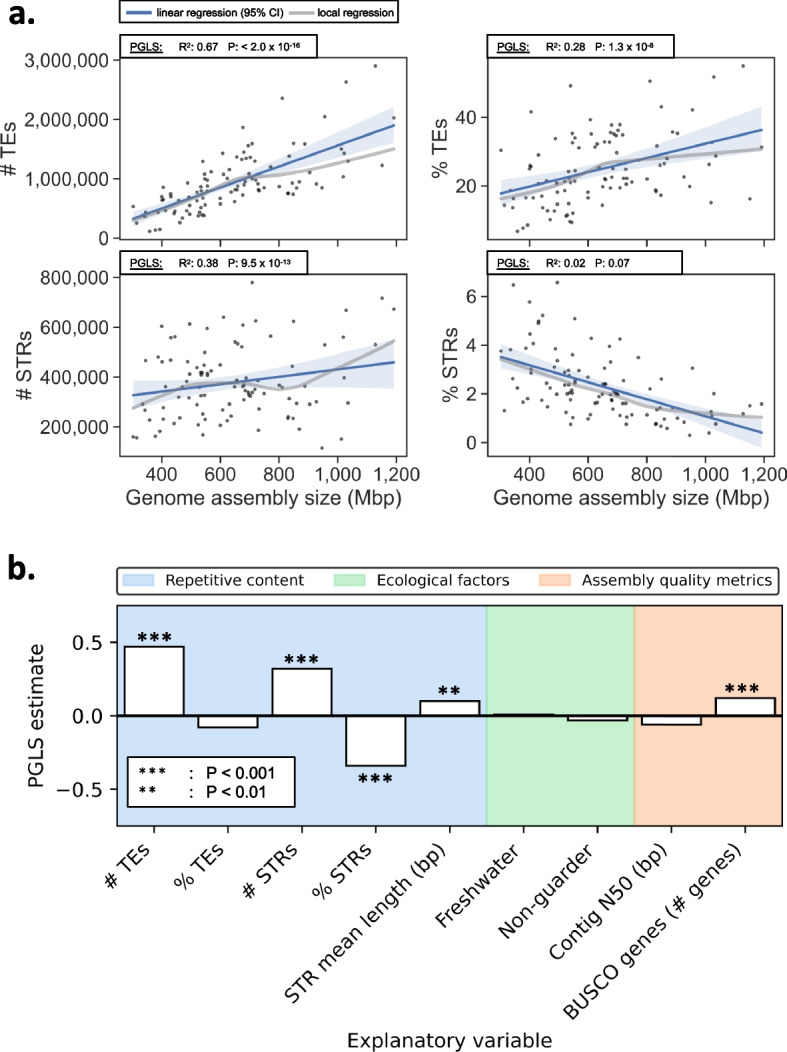
Genome size correlations with repetitive DNA content, ecological variables and genome assembly quality metrics. **a.** Repetitive DNA as a function of genome size. Top left: The number of TEs ("# TEs"). Top right: The percentage of the genome being TEs ("% TEs"). Bottom left: The number of STRs ("# STRs"). Bottom right: The percentage of the genome being STRs ("% STRs"). The blue line and the shaded area indicate a linear regression with the 95% confidence interval, which does not take the correlation between genetic relatedness and the residuals into account. The gray line indicates the local regression. The box above each plot indicates the adjusted R^2^ and the P-value of the fit in PGLS regression. **b**. PGLS regression model with genome assembly size as the response, showing the estimates of the explanatory power of repetitive DNA content, ecological variables and genome assembly quality metrics. The variables were standardized by subtracting the means and divided by the standard deviation. Asterisks indicate that the explanatory variables had significant contributions at the 1% alpha level (**) or 0.1% alpha level (***). See Supplementary Table 2 for model estimates without normalization

We performed phylogenetic generalized least square (PGLS) regression to test if genome assembly size was correlated with the TE and STR content of the assemblies, while taking the phylogenetic relationships among samples into account, as well as the aquatic habitat and degree of parental care. The correlation between the number of TEs and genome assembly size (R^2^: 0.67, *P* < 0.001, Fig. 2a) was stronger than between the genomic proportion of TEs and genome assembly size (R^2^: 0.28, *P* < 0.001, Fig. 2a). The number of STRs displayed a positive correlation with genome size (R^2^: 0.38, *P* < 0.001, Fig. 2a), but the genomic proportion of STRs appeared to have a negative relationship with genome assembly size. The apparent relationship between the genomic proportion of STRs and genomes assembly size did not reach a significance threshold of 5% for a linear relationship in the PGLS model (R^2^: 0.02, *P* > 0.05, Fig. 2a). We omitted the tetraploid species (*Salmo salar* and *Cyprinus carpio*) as well as *Danio rerio* as outliers in terms of assembly quality (Supplementary Table 1, Supplementary Fig. 2). The genomic proportion of TEs did not correlate with the genomic proportion of STRs (R^2^: ~ 0, *P* > 0.1, Supplementary Fig. 3), although the counts correlated (R^2^: 0.21, *P* < 0.001, Supplementary Fig. 3). Next, we treated genome size as a response to TE content, STR content (including the mean length of STRs), aquatic habitat (marine/freshwater), and the degree of parental care in a multiple PGLS regression. To control for differences in assembly quality, we included assembly quality metrics (N50 contig and BUSCO gene completeness, see Supplementary Table 1) as covariates. We included data on aquatic habitat and parental care from FishBase [44], where the degree of parental care was defined according to Balon [45] (Fig. 1). We grouped fish that carries eggs in their mouth or body (bearers) and that guard their eggs in nests or similar (guarders) together. In the full model, TE counts, STR counts, STR mean lengths, and BUSCO gene completeness, had positive correlations with genome assembly size (Fig. 2b, Supplementary Table 2). The genomic proportions of STRs, however, were negatively correlated to genomic assembly size (Fig. 2b, Supplementary Table 2). Contig N50 was not a significant explanatory variable in the model. Together, the variables explained 87% of the variation in the genome assembly sizes observed in our samples.

### STR variation across teleost lineages linked to aquatic habitat

**Fig. 3:**
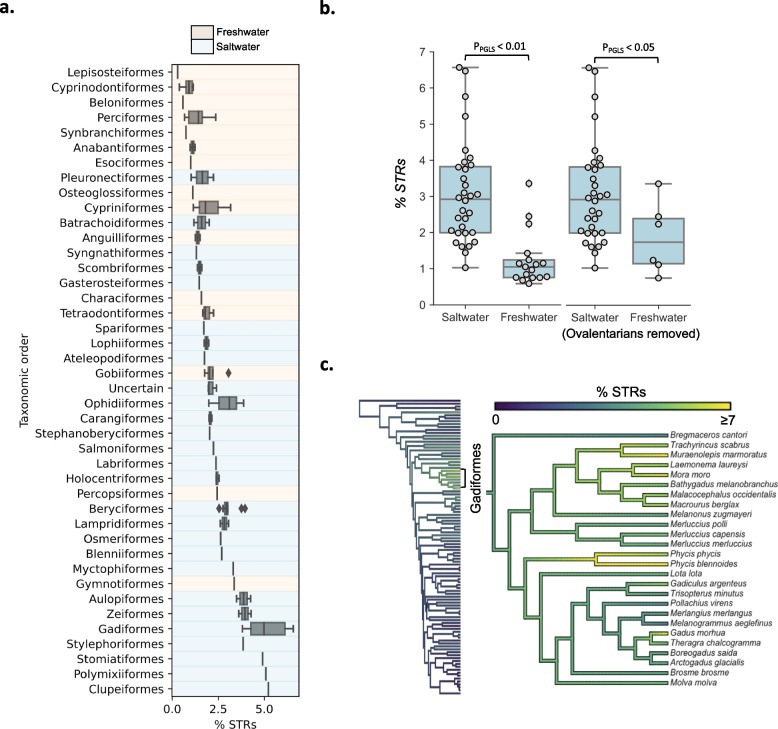
STR content in freshwater fish, saltwater fish, and codfish. **a**. The boxplots show the variation in STR proportions ("% STRs") across the taxonomic orders included in this study. **b**. The boxplots show the genomic proportion of STRs in fish genomes ("% STRs") grouped by saltwater and freshwater. The significance of the difference in the genomic proportion of STRs between freshwater fish and saltwater fish in PGLS regressions are shown above the boxplots. The boxplots to the right show the results when ovalentarian fish were removed (see Fig. 1). **c**. The STR content in Gadiformes (codfish). The phylogenetic tree to the left includes the 101 species from Fig. 1 and is shown with Gadiformes highlighted. The phylogenetic tree to the right show additional codfish species (a subset of the phylogenetic tree from Malmstrøm et al. [41]). The coloring, ranging from blue to yellow, are scaled with the genomic proportion of STRs ("% STRs"), and capped at 7% for clarity

Our STR analyses showed that there is high variability in STR content within teleost fish, both with respect to total STR content and relative differences of STRs with different unit sizes (Fig. 1). One striking pattern is the proportion of STRs with unit size 5–10 in Gobiidae (*Chatrabas melanurus*, *Lesueurigobius cf. sanzoi*, *Periophthalmus magnuspinnatus*, *Periophthalmodon schlosseri*, *Scartelaos histophorus* and *Boleophthalmus pectinirostris*), more specifically decanucleotide repeats. Suspecting that this might be an artifact, we looked at Gobiidae tandem repeats with unit sizes from 1 to 20, and found that the high proportion of decamers (mean: 0.7%, SD: 0.2%) represents a high proportion of k-mers with unit sizes 10–20 (mostly 11-mers), which likely confuses the repeat detection algorithm (Phobos) when repeats are interrupted. Why Gobiidae had such a unique STR landscape (i.e., a relative high abundance of STRs with larger unit sizes) compared to other teleosts requires further investigation. PGLS regression revealed a significant elevation of STR proportions in saltwater fish compared to freshwater fish (P: 0.003, Fig. 3a, b), supporting the tendency found in Yuan et al. [12]. The association was robust to removal of the whole Ovalentaria clade, which mainly contain freshwater fish (Fig. 3b, the ovalentarians are highlighted in Fig. 1). We noted that codfish (Gadiformes) genomes had particularly high proportions of STRs compared to the other species (mean: 5.2%, SD: 1.1%, Fig. 3a). By annotating additional codfish assemblies (from [41, 42]) we found that extreme STR propagation is common within this lineage (Fig. 3c, Supplementary Table 3).

### TE proportion displays a weak, negative correlation with net diversification

**Fig. 4:**
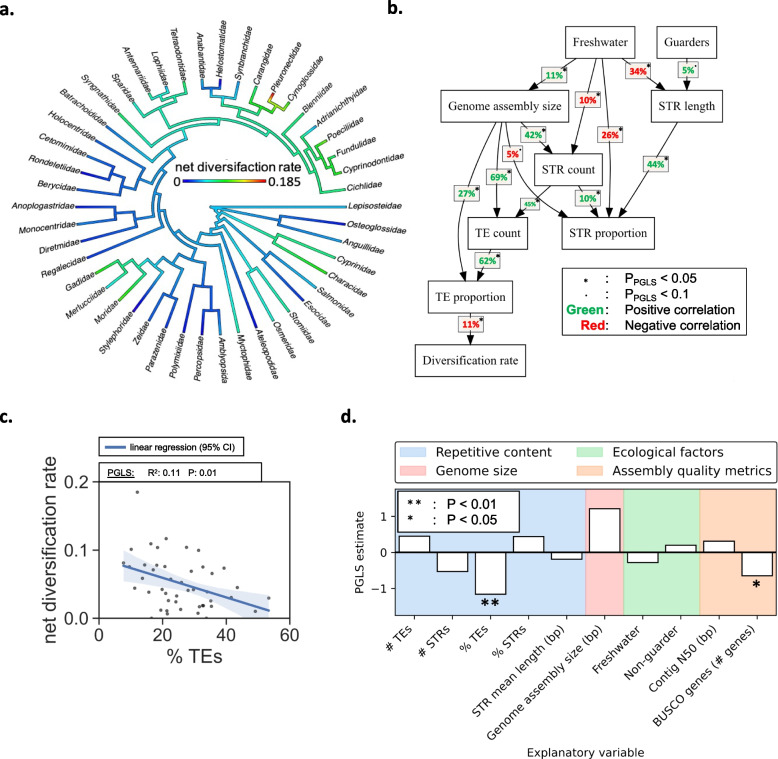
Net diversification rates analyses. **a**. Family-specific net diversification rates as estimated by Scholl and Wiens [46]. **b**. Family-level PGLS regressions between repetitive DNA variables, ecological variables, and genome assembly sizes that reached a 10% alpha-level (*P* < 0.1). **c**. Net diversification rates as a function of the proportion of TEs in the genome. The blue line and the shaded area indicate the regression of a linear model with 95% confidence interval. The box above the plot indicates the result from the PGLS regression. **d**. Multiple PGSL regression model with net diversification rates as a response, showing the PGLS estimates of the variables in b. in addition to the estimates of contig N50 and the number of BUSCO genes. Asterisks indicate that the explanatory variables had significant contributions at the 1% alpha level (**) or at the 5% alpha level (*). See Supplementary Table 5 for model estimates without normalization

We performed a family-level all vs. all PGLS regression to explore the relationship between family-level median values of genome assembly size, repetitive DNA, and the ecological variables (see Fig. 1 for species belonging to the same taxonomic family). To test how these variables correlate with diversification rates, we included net diversification rate estimates from Scholl and Wiens [46], whom calculated diversification rates across the tree of life and included 45 out of 71 of the teleost families surveyed in this study (Fig. 4a, Supplementary Table 4). The family-level all vs. all PGLS regression results indicated that freshwater fish had fewer (R^2^: 0.10, *P* < 0.05) and shorter (R^2^: 0.34, *P* < 0.05) STRs that covered less (R^2^: 0.26, *P* < 0.05) of their slightly larger genomes (R^2^: 0.11, *P* < 0.05), compared to saltwater fish (Fig. 4b). Guarding behavior had a negligible correlation with an increase in the STR length (R^2^: 0.05, *P* < 0.1) (Fig. 4b). Variation in mean STR lengths seemed to explain more variation in STR proportion (R^2^: 0.44, *P* < 0.05) compared to the STR counts directly (R^2^: 0.1, *P* < 0.05) (Fig. 4b). Family-level median genome assembly sizes correlated with both STR counts (R^2^: 0.42, *P* < 0.05) and TE counts (R^2^: 0.69, *P* < 0.05), but in contrast to the species-level regression in the full model shown in Fig. 2b, genome assembly sizes positively correlated with TE proportion (R^2^: 0.27, *P* < 0.05) (Fig. 4b). The negative correlation with STR proportion (Fig. 2a-b) was reiterated, although weakly, in the family-level regression (R^2^: 0.05, *P* < 0.05) (Fig. 4b). The correlation between STR proportion and TE proportion was not significant in PGLS regressions (R^2^: ~ 0, *P* > 0.1), although the counts were correlated (R^2^: 0.45, *P* < 0.05) (Fig. 4b). The PGLS regressions indicated that the proportion of TEs in genomes was the only variable that correlated with family-specific net diversification rates (R^2^: 0.11, *P* < 0.05, Fig. 4b-c). The proportion of TEs was positively correlated to the TE count (R^2^: 0.62, *P* < 0.05) and the genome assembly size (R^2^: 0.27, *P* < 0.05), but neither genome assembly size (R^2^: ~ 0, *P* > 0.1) nor TE count (R^2^: ~ 0, *P* > 0.1) correlated with net diversification rates (Fig. 4b). Next, we tested if the TE proportion had explanatory power in a multiple PGLS regression model including all the above-mentioned variables, and included genome assembly quality metrics: Contig N50 and the number of BUSCO genes. The full PGLS model had an adjusted R^2^ of 0.16 and pointed to TE proportion and the number of BUSCO genes as significant explanatory variables (TE proportion *P* < 0.01, BUSCO genes *P* < 0.05), both with negative PGLS estimates (Fig. 4d). Tests were repeated using net diversification rates based on different assumed extinction rates (0.1, 0.5 and 0.9), which had negligible impacts on the results. The removal of Pleuronectidae, which in our dataset is an outlier in terms of net diversification rate (Fig. 4a, c), led to a drop in the R^2^ of the model by 5% (R^2^: 0.11), the number of BUSCO genes lost significance (P: 0.69), and weakened the statistical significance of TE proportion (P: 0.05).

## Discussion

Using a time-calibrated phylogeny we have investigated the genomic repeat landscape across the teleost radiation. Overall, TE content was not positively associated with net diversification, but significantly contributes to genome size variation. High STR content was associated with smaller genomes, marine habitat and could be linked to high fecundity (such as codfish and Atlantic herring). The proportion of STRs covering the genomes did not correlate with the proportion of TEs in teleost lineages (Supplementary Fig. 3), pointing towards independent evolutionary paths for these types of repeats.

Our results on the contribution of TEs to genome size variation (Fig. 2a, b) support the general tendency observed in chordates [8], vertebrates [7] and previous studies of teleosts [12]. We observed, however, a large variance in our dataset, resulting in fairly low R^2^ of 0.28 (Fig. 2a). This shows that in teleosts, differential abundance of TEs alone could explain 28% of the variation in genome size, when the phylogenetic relationship between samples is taken into account. The larger model that included TE and STR counts, STR lengths, as well as TE and STR proportions, genome assembly quality metrics, habitat, and parental care, which previously have been linked to genome size differences in teleosts [18], explained 87% of the genome size differences in our samples. Given that the extent of parental care and egg size are positively correlated [47], and egg size is positively correlated with genome size [18], we expected to find a positive correlation between parental care and genome size. Contrary to expectation, non-guarding behavior did not have a significant correlation with genome size (Fig. 2b). Further, a marine environment did not explain any difference in genome size when phylogenetic relationships were taken into account (Fig. 2b).

In comparison, STR content was significantly higher in marine fish (Fig. 3a), with the most extreme being the codfish (Fig. 3b). Given the current understanding of STRs as hypervariable regions with occasional functional impact, we speculate that marine species with high fecundity and high mortality of eggs [48], more robustly tolerate the mutational load of STRs, which is likely substantial. Theory predicts [49, 50] that the number of offspring an individual on average needs to produce to keep the population size constant is a function of the deleterious mutation rate and the number of functional mutable sites. It is likely that STRs increase the deleterious mutation rate, although it would depend on the STR mutation rate and the fraction of STRs in functional regions. This could serve as an explanation for why we see elevated STR propagation in marine clades, i.e., fish with higher number of eggs per spawning event, compared with freshwater fish. In particular, for the species with available fecundity estimates (scaled for body size), *G. morhua* and *C. harengus* have the highest fecundity in our dataset [51] and also stand out as having high STR content. However, the only (close to negligible) correlation between parental care and repeat content was between the mean length of STRs and guarding-behavior (Fig. 4b). Improvements in the sequencing and gene annotation of teleost genome assemblies would be required to accurately capture and quantify TEs and STRs present in functional regions of genomes for this hypothesis to be addressed more directly.

We show that DNA transposons are the most common TEs in teleosts (Fig. 2), confirming the pattern observed in other studies. Overall, variation is high across lineages and indicates substantial TE activity over 270 million years of evolution. As elevated TE activity has been shown to coincide with teleost species radiations, such as in salmonids [24] and cichlids [25, 26], and in light of the ongoing discussion of the role of TEs in evolution [52–54], a main objective of this study was to investigate if clades with high TE content have had comparably high net diversification. The test relies on the assumption that a fish family with a high count or high proportion of repetitive elements in their genomes is likely to have had more propagation of repetitive elements than a fish family with a low count or low proportion. Our results do not support that high TE content is linked to higher net diversification rates, but rather show weak support for the contrary (Fig. 4c, d), and we see no apparent pattern with regard to the effect of STRs or genome size, parental care, or aquatic habitat, at least across our broad selection of teleostean families. This does not rule out that TE insertions can lead to novel adaptive traits, and might facilitate diversification in certain teleost clades, as indicated in studies of African cichlids [25, 26, 55]. However, a general speciation promoting role for TEs is not reflected in our results.

Throughout the study, we assessed TE content to be the sum of the interspersed repetitive elements judged by our tools (BLAST, BLASTX and HMMR) to be a TE in addition to interspersed repetitive elements not successfully classified. The classification process is limited by the extent of prior annotated TEs, which in teleosts are biased towards *D. rerio*. This is illustrated by the values obtained from zebrafish that has the most extensive prior annotation, and the percentage of classified TEs (48.0%) is very close to the total interspersed repeats (52.2%, Supplementary Table 1), which is not the case for most other surveyed fish (Supplementary Fig. 1a). The detection of interspersed repeats is not biased by a priori information, but can be influenced by assembly quality [56, 57]. However, in our models that include multiple covariates, we found that the common assembly quality metric; contig N50 did not impact our conclusions. Gene completeness as measured by the number of detected BUSCO genes did however explain statistically significant amounts of variation in our models (Fig. 2b, Fig. 4d). Thus, a portion of the genome size variation we observe in our samples could be explained by assembly quality, which highlights the need for high-quality teleost genomes.

It should further be noted that genomes inhabiting high numbers of identical TEs (i.e., families that recently expanded) are expected to be harder to assemble, as identical sequences create collapsed repeats. This can lead to an underreporting of elements in genomes with recent expansions. It is also known that high STR content in combination with short read sequencing can produce assemblies of lower quality, as reported in the sequencing efforts of the Atlantic cod (*G. morhua*) genome [32]. This implies that assemblies with low assembly quality likely are underestimated with regards to STR content.

Regardless of some limitations, our results suggest that high proportions of TEs are not positively correlated with net diversification rates in teleost clades, and that elevated levels of STRs are linked to and must thus be tolerated by marine teleosts, potentially due to higher fecundity. Such a link would be very important for understanding genome evolution, but needs to be further investigated within teleosts, as well as in other organism groups.

## Material and methods

### Genome assemblies and phylogenies

Fifty-six genome assemblies were retrieved from a teleost genome data release [42], and 10 assemblies were sequenced and assembled by [43], which also released the 101-species phylogeny. The remaining 46 genome assemblies were retrieved from ENSEMBL and NCBI. For an overview of assembly origins, see [43] and Supplementary Table 1. The codfish phylogeny was taken from [41]. Details regarding the phylogeny construction can be found in these respective studies.

### TE and STR annotation

For TE annotation, we used a variant of the computational pipeline that is more thoroughly described in [32], available at https://github.com/uio-cels/Repeats. The pipeline includes multiple TE detection steps using different tools, steps for removing non-TEs from the detected sequences and steps for classifying the elements. For the initial detection step, we used RepeatModeler (v. 1.0.8) [58] and LTRharvest (part of GenomeTools v. 1.5.7) [59]. RepeatModeler detects all sorts of repetitive sequences and LTRharvest is specialized for detecting LTR-RTs. Using BLASTX, TEs with sequences matching known non-TEs in UniProtKB/Swiss-Prot were removed. To classify the TEs, we used RepeatClassifier, which is a part of the RepeatModeler software. As the tool did not manage to classify all of the remaining sequences, additional similarity searches were performed between the sequences and a curated library of TE sequences (RepBase v. 20,150,807), using nucleotide BLAST. Finally, we built Hidden Markov Model profiles from the detected sequences using HMMER (v. 3.1b1) [60] and compared the profiles with HMM profiles from databases downloaded from GyDB.org [61] and dfam.org [62], using the nhmmer feature included in HMMER. This resulted in additional sequences being classified at the class and subclass level. The pipeline resulted in one de novo library per assembly, which contained the consensus sequences of the interspersed repeats detected in each assembly. We merged the de novo TE library with a library of known eukaryotic TEs (RepBase) and used this as input for RepeatMasker (v. 4.0.6), run with the -s (sensitive) option. The.out and.tbl files produced by RepeatMasker served as the basis for the downstream analysis, performed using custom Python scripts. For detection of STRs we used Phobos v3.3.12 [16] to detect all STRs with unit size 1–10 bp in the genome assemblies. The output was in Phobos native format which was further processed with the sat-stat v1.3.12 program, yielding files with statistics and a GFF file. Other options were set as in Tørresen et al. [32]. For the Gobiidae genomes, we ran Phobos with unit sizes 1–20 bp. To compare assembly-based TE annotation with read-based TE annotation 53 assemblies were re-analyzed with de novo assembly & annotation Pipeline for Transposable Elements (dnaPipeTE), which detects and annotates TEs from raw reads [63].

### Diversification rates

We retrieved estimates of net diversification rates from Scholl and Wiens [46], who calculated diversification rates based on the stem ages of teleost families from the teleost phylogenetic tree produced by Betancur-R et al. [64]. They used the method-of-moments estimator as described by Magallon and Sanderson [65],

1 $$r=\frac{1}{t}log\left(n\left(1 -\varepsilon \right)+\varepsilon \right)$$

where $r$ is the net diversification rate estimate, $t$ is the family stem age, $n$ is the number of extant species and $\varepsilon$ is the relative extinction rate. $\varepsilon$ is included to correct for unsampled, extinct clades. The estimates used in this study are based on the $r$ values when $\varepsilon$ was set to 0.1, 0.5 and 0.9. Note that more recent diversification estimates are available [66], but cover only marine fish.

### Comparative phylogenetic analyses

Statistical analysis was performed using phylogenetic least-squares (PGLS) regressions using the R package ‘caper’ v. 1.1.0 [67]. PGLS is a commonly used method for incorporating phylogenetic information in the modelling of associations between traits. PGLS assumes that more closely related species have more similar traits and uses the expected covariance structure to modify the slope and intercept estimates. For tests with net diversification rates, we used a pruned phylogeny containing tips representing teleost family stem ages, and used median values per family for all covariates. In all tests, we optimized branch length transformations using maximum likelihood. LOWESS (locally weighted linear regressions) lines were created using the ‘seaborn’ Python package with the ‘regplot’ function and standard parameters.

### Gene completeness analysis

We counted how many of 3,698 highly conserved acanthopterygian genes that were present in each assembly, estimated by the "BUSCO complete single" count generated from Benchmarking Universal Single-Copy Orthologs (BUSCO) v. 1.1b [68], which was run on each assembly. The "BUSCO completed duplicated" counts were used to indicate ploidy.

### Supplementary Information

**Additional file 1: Supplementary Table 1, 3, 4.** Sample information and the data used in this study (XLS).

**Additional file 2: Supplementary Table 2.** PGLS regression estimates with genome size as a response, without normalizing the explanatory variables, relevant to Fig. 2b. **Supplementary Table 5.** PGLS regression estimates with net diversification rates as a response, without normalizing the explanatory variables, relevant to Fig. 4d. **Supplementary Figure 1.** a. The percentage of interspersed elements in teleost genomes classified as transposable elements (TEs) ("% Classified as TEs"), as a function of the percentage of classified and unclassified interspersed elements ("% Classified and unclassified interspersed elements"). b. Comparison between the % of interspersed elements detected with assembly-based detection and annotation methods (Repeatmodeler, y-axis) and read-based detection and annotation methods for 53 teleost genomes (dnaPipeTE, x-axis). **Supplementary Figure 2.** Genome size regressions with repetitive DNA content as in Figure 2a, *D. rerio*, *C. carpio*, and *S. salar* are included. **Supplementary Figure 3.** Left: The percentage of transposable elements (TEs) in teleost genomes ("%TEs", y-axis) as a function of the percentage of short tandem repeats ("% STRs"). Right: The count of TEs in teleost genomes as a function of the count of STRs.

## Data Availability

Summaries of the annotation of TEs and STRs, along with the ecological data, are in Supplementary Table 1. Species-specific annotations of TEs and TE-derived DNA can be found at: https://doi.org/10.6084/m9.figshare.8280800 (~ 4.6 Gb). The R script used for statistical analysis, can be found at https://github.com/uio-cels/teleost-repeats. The TE consensus sequences generated from each assembly can be found at https://doi.org/10.5061/dryad.4xgxd25g9.
