# Supplementary material for: Teleost genomic repeat landscapes in light of diversification rates and ecology
Source: Mob DNA. 2023 Oct 3;14:14. doi: 10.1186/s13100-023-00302-9 (PMC10546739; doi:10.1186/s13100-023-00302-9)
Supplement: Supplementary file 2 — Additional file 2: Supplementary Table 2. PGLS regression estimates with genome size as a response, without normalizing the explanatory variables, relevant to Fig. 2b. Supplementary Table 5. PGLS regression estimates with net diversification rates as a response, without normalizing the explanatory variables, relevant to Fig. 4d. Supplementary Figure 1. a. The percentage of interspersed elements in teleost genomes classified as transposable elements (TEs) ("% Classified as TEs"), as a function of the percentage of classified and unclassified interspersed elements ("% Classified and unclassified interspersed elements"). b. Comparison between the % of interspersed elements detected with assembly-based detection and annotation methods (Repeatmodeler, y-axis) and read-based detection and annotation methods for 53 teleost genomes (dnaPipeTE, x-axis). Supplementary Figure 2. Genome size regressions with repetitive DNA content as in Figure 2a, D. rerio, C. carpio, and S. salar are included. Supplementary Figure 3. Left: The percentage of transposable elements (TEs) in teleost genomes ("%TEs", y-axis) as a function of the percentage of short tandem repeats ("% STRs"). Right: The count of TEs in teleost genomes as a function of the count of STRs. [file 13100_2023_302_MOESM2_ESM.docx]

**Supplementary Tables and Figures**

**Supplementary Table 1, 3, 4.** Sample information and the data used in this study (XLS).

**Supplementary Table 2.** PGLS regression estimates with genome size as a response, without normalizing the explanatory variables, relevant to Figure 2b.

| **variable** | **Estimate (bp)** | **Std. Error** | **T-value** | **P-value** |
| --- | --- | --- | --- | --- |
| intercept | 310,520,000 | 67,244,000 | 4.6 | 1.3 x 10^-5^ |
| # TEs | 256.9 | 32.3 | 8.0 | 5.5 x 10^-12^ |
| % TEs | -2,299,500 | 1,566,600 | -1.5 | 0.15 |
| # STRs | 555.5 | 100.2 | 5.5 | 3.1 x 10^-7^ |
| % STRs | -81,095,000 | 15,473,000 | -5.2 | 1.1 x 10^-6^ |
| STR length | 3,382,300 | 1,198,000 | 2.8 | 0.006 |
| Freshwater | 5,802,100 | 14,538,000 | 0.40 | 0.69 |
| Non-guarder | -23,469,000 | 17,428,000 | -1.3 | 0.18 |
| Gene completeness | 41,728 | 9,444.8 | 4.4 | 2.8 x 10^-5^ |
| Contig N50 | -187.8 | 278.12 | -0.68 | 0.50 |

**Supplementary Table 5.** PGLS regression estimates with net diversification rates as a response, without normalizing the explanatory variables, relevant to Figure 4d.

| **variable** | **Estimate (species/myr)** | **Std. Error** | **T-value** | **P-value** |
| --- | --- | --- | --- | --- |
| intercept | 0.16 | 0.06 | 2.5 | 0.02 |
| # TEs | 2.8 x 10^-8^ | 3.6 x 10^-8^ | 0.79 | 0.43 |
| % TEs | -0.0042 | 0.0013 | -3.2 | 0.003 |
| # STRs | -1.0 x 10^-7^ | 8.8 x 10^-8^ | -1.14 | 0.26 |
| % STRs | 0.012 | 0.013 | 0.92 | 0.37 |
| STR length | -0.0007 | 1.2 x 10^-3^ | -0.63 | 0.53 |
| Freshwater | -0.022 | 0.02 | -1.1 | 0.29 |
| Non-guarder | 0.02 | 0.014 | 1.3 | 0.19 |
| Gene completeness | -2.7 x 10^-5^ | 1.1 x 10^-5^ | -2.3 | 0.03 |
| Contig N50 | 7.5 x 10^-7^ | 5.9 x 10^-7^ | 1.27 | 0.21 |

**Supplementary Figure 1.** **a.** The percentage of interspersed elements in teleost genomes classified as transposable elements (TEs) ("% Classified as TEs"), as a function of the percentage of classified and unclassified interspersed elements ("% Classified and unclassified interspersed elements"). **b.** Comparison between the % of interspersed elements detected with assembly-based detection and annotation methods (Repeatmodeler, y-axis) and read-based detection and annotation methods for 53 teleost genomes (dnaPipeTE, x-axis).

**Supplementary Figure 2.** Genome size regressions with repetitive DNA content as in Figure 2a, *D. rerio*, *C. carpio*, and *S. salar* are included.

**Supplementary Figure 3.** Left: The percentage of transposable elements (TEs) in teleost genomes ("%TEs", y-axis) as a function of the percentage of short tandem repeats ("% STRs"). Right: The count of TEs in teleost genomes as a function of the count of STRs.
